# Detection of *Schistosoma mansoni*-derived DNA in human urine samples by loop-mediated isothermal amplification (LAMP)

**DOI:** 10.1371/journal.pone.0214125

**Published:** 2019-03-26

**Authors:** Pedro Fernández-Soto, Javier Gandasegui, Cristina Carranza Rodríguez, José Luis Pérez-Arellano, Beatriz Crego-Vicente, Juan García-Bernalt Diego, Julio López-Abán, Belén Vicente, Antonio Muro

**Affiliations:** 1 Infectious and Tropical Diseases Research Group (e-INTRO), Biomedical Research Institute of Salamanca-Research Centre for Tropical Diseases at the University of Salamanca (IBSAL-CIETUS), Faculty of Pharmacy, University of Salamanca, Salamanca, Spain; 2 Department of Medical and Surgical Sciences, University of Las Palmas de Gran Canaria, Las Palmas de Gran Canaria, Spain; 3 Unit of Infectious Diseases, Maternal and Child Insular University Hospital Complex, Las Palmas de Gran Canaria, Spain; Universidad Nacional Autonoma de Mexico, MEXICO

## Abstract

**Background:**

*Schistosoma mansoni* is the main species causing hepatic and intestinal schistosomiasis in Sub-Saharan Africa, and it is the only species in South America. Adult stages of the parasite reside in the mesenteric venous plexus of infected hosts, and eggs are shed in feces. Collecting patient stool samples for *S*. *mansoni* diagnostic purposes is difficult in large-scale field trials. Urine samples would be an alternative approach for molecular *S*. *mansoni* detection since they have several advantages over stool samples, including better handling, management and storage. Additionally, loop-mediated isothermal amplification (LAMP) technology is a powerful molecular diagnostic tool for infectious diseases, particularly under field conditions in developing countries. The present study aimed to assess the effectiveness of our previously developed LAMP assay (SmMIT-LAMP) for *S*. *mansoni*-specific detection in clinical urine samples.

**Methodology/Principal findings:**

The sensitivity of SmMIT-LAMP in urine was established in simulated fresh human urine samples artificially spiked with genomic DNA from *S*. *mansoni*. LAMP for 120 min instead of 60 min improved the sensitivity, reaching values of 0.01 fg/μL. A set of well-defined frozen stored human urine samples collected from Sub-Saharan immigrant patients was selected from a biobank to evaluate the diagnostic validity of SmMIT-LAMP. The set included urine samples from patients with microscopy-confirmed infections with *S*. *mansoni*, *S*. *haematobium* and other nonschistosome parasites, as well as urine samples from patients with microscopy-negative eosinophilia without a confirmed diagnosis. The SmMIT-LAMP was incubated for 60 and 120 min. A longer incubation time was shown to increase the LAMP-positive results in patient urine samples. We also tested urine samples from mice experimentally infected with *S*. *mansoni*, and LAMP-positive results were obtained from the third week after infection. A real-time LAMP assay was also performed with three individual urine samples.

**Conclusions/Significance:**

The SmMIT-LAMP could effectively detect *S*. *mansoni* DNA in mouse urine samples and produced promising results for human clinical samples. The detection of *S*. *mansoni* DNA in mouse urine samples from the third week after infection indicates that early diagnosis of active *S*. *mansoni* infection is possible using urine as a source of DNA. Further studies are still needed, but our method could be used as a promising molecular tool applicable to urine samples to diagnose human intestinal schistosomiasis caused by *S*. *mansoni*.

## Introduction

Human schistosomiasis, a parasitic disease caused by several species of trematode flatworms of the genus *Schistosoma*, is an endemic disease in 74 countries, infecting more than 200 million people worldwide [1]. *Schistosoma mansoni* is the main species causing hepatic and intestinal schistosomiasis in Sub-Saharan Africa, and it is the only species in South America [1–4]. The prevalence of imported schistosomiasis is growing in nonendemic areas due to the increase in international travelers to endemic areas, as well as expatriates and immigrants from endemic countries [5–7].

Different diagnostic techniques are used in diagnosing schistosomiasis. The traditional Kato-Katz (KK) fecal microscopic examination for counting schistosome eggs and immunology-based analyses detecting schistosome-derived circulating anodic (CAA) and cathodic (CCA) antigens mainly lack sensitivity in low-intensity infections and posttreatment conditions [8, 9]. Antibody detection also lacks specificity, causing a high level of cross-reactivity [9]. Nonetheless, the detection of CAA and CCA has a number of potential advantages over specific antibody detection because both can be detected in urine [9, 10]. Today, new technologies and better diagnostic tests for schistosomiasis in field conditions in endemic areas, as well as for routine diagnosis in developed countries, are still needed.

Collection of samples for diagnostic purposes, such as stools, blood or tissue biopsies, can be quite difficult in some population groups. Urine is a biological sample that would have potential advantages in the detection of *S*. *mansoni* over other samples, as it can be collected noninvasively (*versus* blood and biopsies), and large quantities of it can easily be obtained. In addition, it is more convenient to collect from patients of all ages, and it is associated with greater ease in terms of handling, management and storage than blood and stool samples. Small amounts of cell-free DNA (cfDNA) in excreted urine can be detected, representing a potentially useful source of genetic material for diagnostic applications [11–13]. Additionally, the utility of detecting parasite-specific DNA in human urine samples has already been demonstrated for schistosomes by a number of conventional and real-time PCR (qPCR)-based methods [14–20, 21] and loop-mediated isothermal amplification (LAMP) assays [22, 23].

The LAMP assay was described by Notomi *et al*. [24] as a rapid, sensitive, specific and time-efficient gene amplification technique that amplifies nucleic acids under isothermal conditions, typically using four different primers designed to recognize six distinct regions on the target sequence. Many salient advantages of LAMP over most PCR-based technologies have been reported [25]. LAMP technology shows all of the characteristics required for potential use in clinical diagnosis and surveillance of infectious diseases, particularly under field conditions in developing countries for most Neglected Tropical Diseases (NTDs), including schistosomiasis [26, 27].

In a previous work, we developed a sensitive and specific LAMP assay (named SmMIT-LAMP) for the successful early detection of *S*. *mansoni* DNA in stool samples from a mouse model of schistosomiasis [28]. In this study, we examine the application of SmMIT-LAMP for urine in a mouse model of *S*. *mansoni* and in human urine samples, both spiked and naturally infected.

## Materials and methods

### Ethics statement

The study protocol was approved by the institutional research commission of the University of Salamanca. Ethical approval was obtained from the Ethics Committee of the University of Salamanca (protocol approval number 48531). Animal procedures in this study complied with the Spanish (Real Decreto RD53/2013) and the European Union (European Directive 2010/63/EU) guidelines on animal experimentation for the protection and humane use of laboratory animals and were conducted at the accredited Animal Experimentation Facility (Servicio de Experimentación Animal) of the University of Salamanca (Register number: PAE/SA/001). Human urine samples used in this study were obtained as part of public health diagnostic activities at Hospital Universitario Insular, Las Palmas de Gran Canaria, Gran Canaria, Spain, and all were obtained under written informed consent and were coded and tested as anonymous samples. Participants were given detailed explanations about the aims, procedures and possible benefit of the study. A standardized epidemiological questionnaire and clinical information were obtained from each participant included in the study. Later, samples were sent and stored at CIETUS, University of Salamanca, Spain, for further molecular analysis. The participation of healthy urine donors (lab staff) for obtaining simulated artificial urine samples and negative urine samples was voluntary. Participants received written and verbal descriptions of the experiment and provided written informed consent prior to testing.

### Urine sample collection

#### Patient urine samples

A total of 28 human urine samples were selected from a set of frozen samples stored at the CIETUS-urine biobank. These urine samples were collected from Sub-Saharan immigrant patients attending during May 2002 to April 2009 at Hospital Universitario Insular, Las Palmas de Gran Canaria, Spain, as part of public health diagnostic activities. While attending, several parasitological diagnostic tests for suspected infectious diseases, such as for schistosomiasis, were performed according to standard routine laboratory procedures. Urine was collected and frozen prior to receiving treatment. The selected set of 28 frozen urine samples was divided into 4 groups as follows: group 1 (n = 7), urine samples from patients with confirmed schistosomiasis caused by *S*. *mansoni* through the detection of parasite eggs in stools by the KK technique; group 2 (n = 7), urine samples from patients with confirmed schistosomiasis caused by *S*. *haematobium* through the detection of parasite eggs in urine by sedimentation or filtration methods; group 3 (n = 7), urine samples from patients with a microscopy-confirmed infection with other nonschistosome parasites; group 4 (n = 7), urine samples from patients with eosinophilia without a confirmed diagnosis and negative on all parasitological tests. Table 1 shows the selected groups of urine samples included in this study. Additionally, fresh urine from a healthy volunteer staff donor with no history of travel to endemic areas of schistosomiasis was collected to use as a negative control.

**Table 1:** Patient urine samples included in the study. Sample groups, sample numbers, origin of the patients, presence/absence of eosinophilia, parasitological finding based on microscopy test and LAMP results for *Schistosoma mansoni* detection are indicated.

Groups	Parasitological finding	No.	Origin	Eosinophilia	LAMP
G1 (n = 7)	*Schistosoma mansoni*	204	Mali	yes	Positive
		205	Mali	yes	Negative
		207	Cameroon	yes	Positive
		249	Mali	no	Positive
		259	Unknown	no	Negative
		339	Liberia	yes	Positive
		54	Mauritania	no	Positive
G2 (n = 7)	*Schistosoma haematobium*	01	Mali	no	Negative
		04	Mali	no	Negative
		06	Mali	yes	Negative
		09	Mali	no	Negative
		17	Mali	no	Negative
		90	Mali	no	Negative
		92	Mali	yes	Negative
G3 (n = 7)	*Hookworms*, *Giardia intestinalis*	214	Cameroon	no	Negative
	*Strongyloides stercoralis*	218	Ghana	no	Negative
	*Plasmodium falciparum*	236	Cameroon	no	Negative
	*Trichomonas vaginalis*	237	Nigeria	no	Positive
	*Enterobius vermicularis*	354	Morocco	yes	Negative
	*Loa loa*, *Mansonella* spp., *Trichuris trichiura*, *hookworms*	416	Equatorial Guinea	yes	Negative
	*Trichuris trichiura*	427	Ivory Coast	no	Positive
G4 (n = 7)	Negative	250	Cape Verde	yes	Positive
		256	Nigeria	yes	Negative
		260	Ghana	yes	Negative
		365	Unknown	yes	Positive
		422	Mali	yes	Positive
		444	Ghana	yes	Positive
		488	Ghana	yes	Negative

G1, G2, G3, G4; groups of urine samples from patients with confirmed infection by *Schistosoma mansoni* (G1), by *S*. *haematobium* (G2), by other nonschistosome parasites (G3) and with microscopy-negative eosinophilia without confirmed diagnosis (G4).

### Simulated urine samples

Fresh urine was collected from a healthy staff donor with no history of travel to endemic areas of schistosomiasis to assess both the sensitivity and specificity of the LAMP assay. To test the sensitivity, collected urine was divided into aliquots of 100 μL each and was then spiked with 2 μL of 10-fold serially diluted *S*. *mansoni* DNA ranging from 50 ng/μL to 0.5 ag/μL (see below), thus resulting in a set of simulated urine samples with a final parasite DNA concentration ranging from 1 ng/μL to 0.01 ag/μL. To test the specificity, a set of tubes was prepared, each containing 100 μL of urine and spiked with 0.5 ng of DNA from several available helminths, alone and in combination with *S*. *mansoni*, including *S*. *haematobium*, *S*. *intercalatum*, *S*. *bovis*, *S*. *japonicum*, *Fasciola hepatica*, *Amphimerus* sp. and *Strongyloides venezuelensis*. All simulated urine samples were prepared when required and directly processed for DNA extraction without previous storage.

#### Mouse urine samples

Five-week-old female CD1 mice experimentally infected with *S*. *mansoni* were selected among those routinely maintaining the experimental cycle of the parasite in our laboratory to be used as sources for urine samples. CD1 mice were infected with 150 *S*. *mansoni* cercariae following the methodology previously described by Smithers *et al*. [29]. Uninfected mice were used as sources for urine samples for negative controls.

Animals were housed at the accredited Animal Experimentation Facility of the University of Salamanca in individual metabolic polycarbonate cages and placed in a humidity- and temperature-controlled environment with a 12-h photoperiod and received sterilized food and water *ad libitum*. To collect urine samples, each mouse was removed from the cage and housed individually in a plastic beaker cleaned with 70% v/v ethanol. Then, individual urine samples (approximately 40–100 μL per infected mouse), either found in the plastic beaker or by gently stroking the lower side of the abdomen, were collected by using a pipette and transferred to a 1.5 mL tube. Individual samples were taken from week 0 to week 8 postinfection (p.i.) and were then pooled from each time point in a mix to minimize the number of subsequent reactions. The weekly pooled samples (approximately 0.5–1 mL) were stored at -20°C for further DNA extraction for molecular analyses.

Following the same procedure, individual urine samples from three additional *S*. *mansoni*-infected mice were collected at week 5 p.i. to extract DNA to test SmMIT-LAMP in a real-time assay.

### DNA extraction

#### Parasite DNA samples

*S*. *mansoni* DNA (Brazilian strain) was extracted from frozen adult male and female worms available in our laboratory using a DNeasy Blood & Tissue Kit (QIAGEN, Hilden, Germany) according to the manufacturer's instructions. The concentration of *S*. *mansoni* DNA was measured using a Nanodrop ND-1000 spectrophotometer (Nanodrop Technologies) and was then diluted with ultrapure water to a final concentration of 50 ng/μL. Serial 10-fold dilutions were prepared with ultrapure water ranging from 50 ng/μL to 0.5 ag/μL to prepare the simulated urine samples as mentioned above. *S*. *mansoni* genomic DNA (2 μL; 0.5 ng/μL) was used as a positive control in all LAMP trials. Negative controls were also utilized in the different trials. Other parasite DNA, including *Schistosoma haematobium*, *S*. *intercalatum*, *S*. *bovis*, *S*. *japonicum*, *Fasciola hepatica*, *Amphimerus* sp. (kindly provided by several colleagues) and *Strongyloides venezuelensis* (extracted from infective third-stage larvae from the feces from rats routinely infected with the parasite in our laboratory), were available in our laboratory and were used for specificity trials (2 μL; 0.5 ng/μL).

#### DNA from patient urine samples

After thawing, urine aliquots (1 mL) were taken and then centrifuged at 5,000 rpm for 5 min at RT to pellet the urinary sediment. Excess supernatant was discarded, but a minimal volume of approximately 100 μL was maintained to resuspend the pellet at the bottom of the tube. DNA was extracted from the 100 μL aliquots using the i-genomic Urine DNA Extraction Mini Kit (Intron Biotechnology) following the manufacturer's instructions and was then stored at -20°C until use in LAMP reactions.

#### DNA from simulated urine samples

DNA was extracted from all fresh simulated urine samples using the i-genomic Urine DNA Extraction Mini Kit (Intron Biotechnology, UK) following the manufacturer's instructions. Purified DNA samples were stored at -20°C until use.

#### DNA from mouse urine samples

Frozen weekly pooled urine samples (approximately 100 μL) collected at weeks 0–8 p.i. and fresh individual urine samples (approximately 60 μL) collected *ad hoc* at week 5 p.i. from *S*. *mansoni*-infected mice were used for DNA extraction using the i-genomic Urine DNA Extraction Mini Kit (Intron Biotechnology) following the manufacturer's instructions. Once extracted, purified DNA samples were stored at -20°C until use in molecular analyses.

### LAMP assays

All LAMP reactions were accomplished using the reaction mixture and specific primer set (named SmMIT-LAMP assay) established by our group and previously described elsewhere [28]. Briefly, the reaction was carried out in a volume of 25 μL of reaction mixture containing 40 pmol each of FIP and BIP primers, 5 pmol each of F3 and B3 primers, 1.4 mM of each dNTP, 1x Isothermal Amplification Buffer—20 mM Tris-HCl (pH 8.8), 50 mM KCl, 10 mM (NH_4_)_2_SO_4_, 2 mM MgSO_4_, 0.1% Tween20 (New England Biolabs, UK), 1 M betaine (Sigma, USA), supplementary 6 mM MgSO_4_ (New England Biolabs, UK) and 8 U of *Bst* 2.0 WarmStart DNA polymerase (New England Biolabs, UK) with 2 μL of template DNA. Reaction tubes were placed in a heating block at 65°C for 60 min or 120 min and were then heated at 80°C for 5 min to stop the reaction. The results were assessed by visual detection of fluorescence (green: positive; orange: negative) after adding 2 μL of 1:10 diluted 10,000X concentration SYBR Green I (Invitrogen) to the final volume. To avoid potential cross-contamination with amplified products, the tubes were briefly centrifuged and carefully opened before adding the dye. The LAMP products (3–5 μL) were also monitored using 1.5% agarose gel electrophoresis and were documented with a gel documentation system (UVItec, UK).

LAMP mixes (25 μL) for real-time reactions were prepared as those for SmMIT-LAMP but also including EvaGreen 20X (Biotium) to monitor the amplification. Real-time reactions were performed in 8-tube Genie Strips using the portable Genie III device (Optigene Ltd., Horsham, UK) at 63°C for 60 min, followed by 10 min at 80°C. The results were also confirmed on 1.5% agarose gels.

## Results

### Application of SmMIT-LAMP on spiked human urine samples

**Fig 1:**
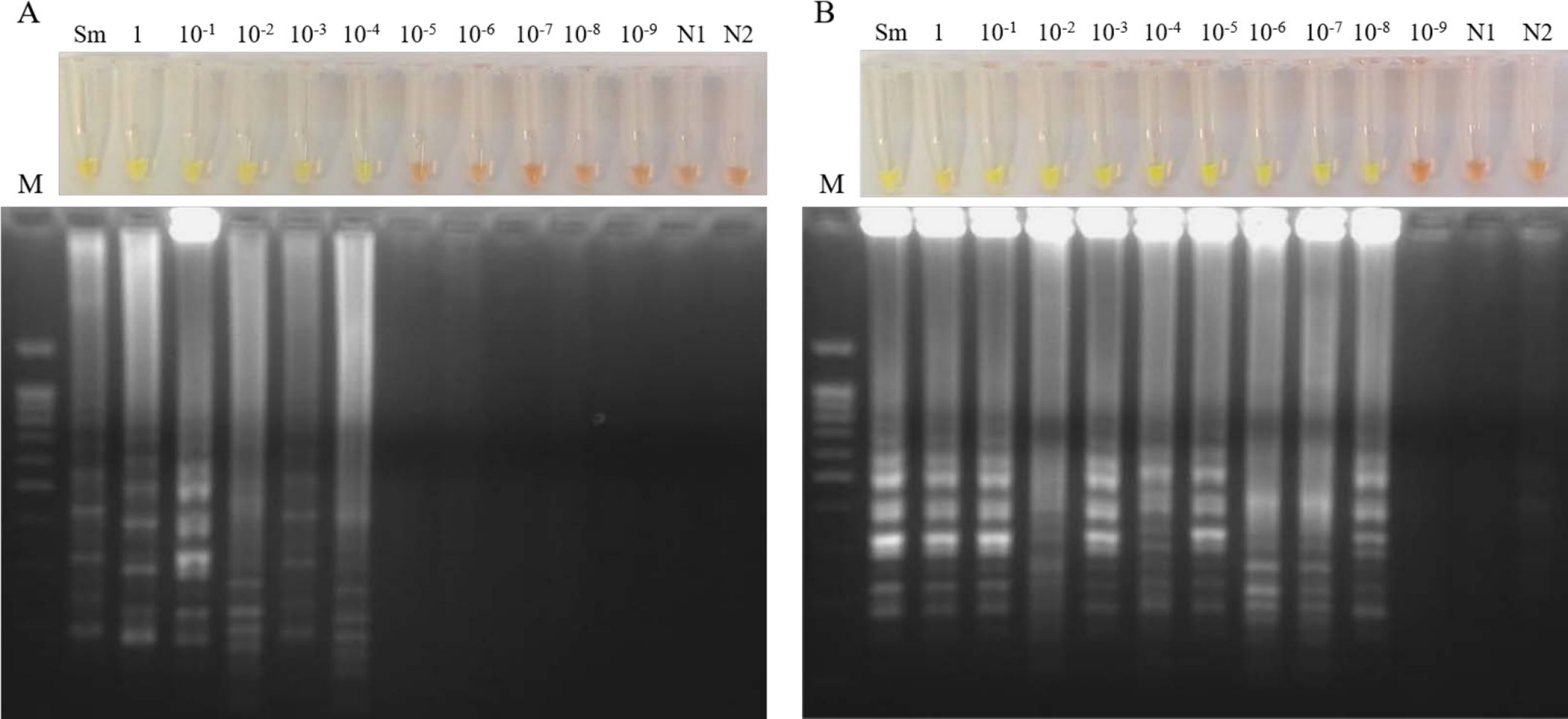
Sensitivity of the SmMIT-LAMP assay in simulated human urine samples. (A) Sensitivity assessment of LAMP when incubating for 60 min. (B) Sensitivity assessment of LAMP when incubating for 120 min. Lanes M: DNA ladder; lanes Sm: genomic DNA from *S*. *mansoni* (1 ng); lanes 1–10^−9^: 10-fold serial dilutions (1 ng/μL to 0.01 ag/μL); lanes N1 and N2: negative controls (no DNA, water as template).

The sensitivity of the LAMP assay in simulated fresh human urine samples artificially spiked with DNA from *S*. *mansoni* is shown in Fig 1. The detection limit of LAMP was 100 fg/μL when incubating the tubes for 60 min (Fig 1A), whereas the detection limit was established at 0.01 fg/μL when incubating for 120 min (Fig 1B).

**Fig 2:**
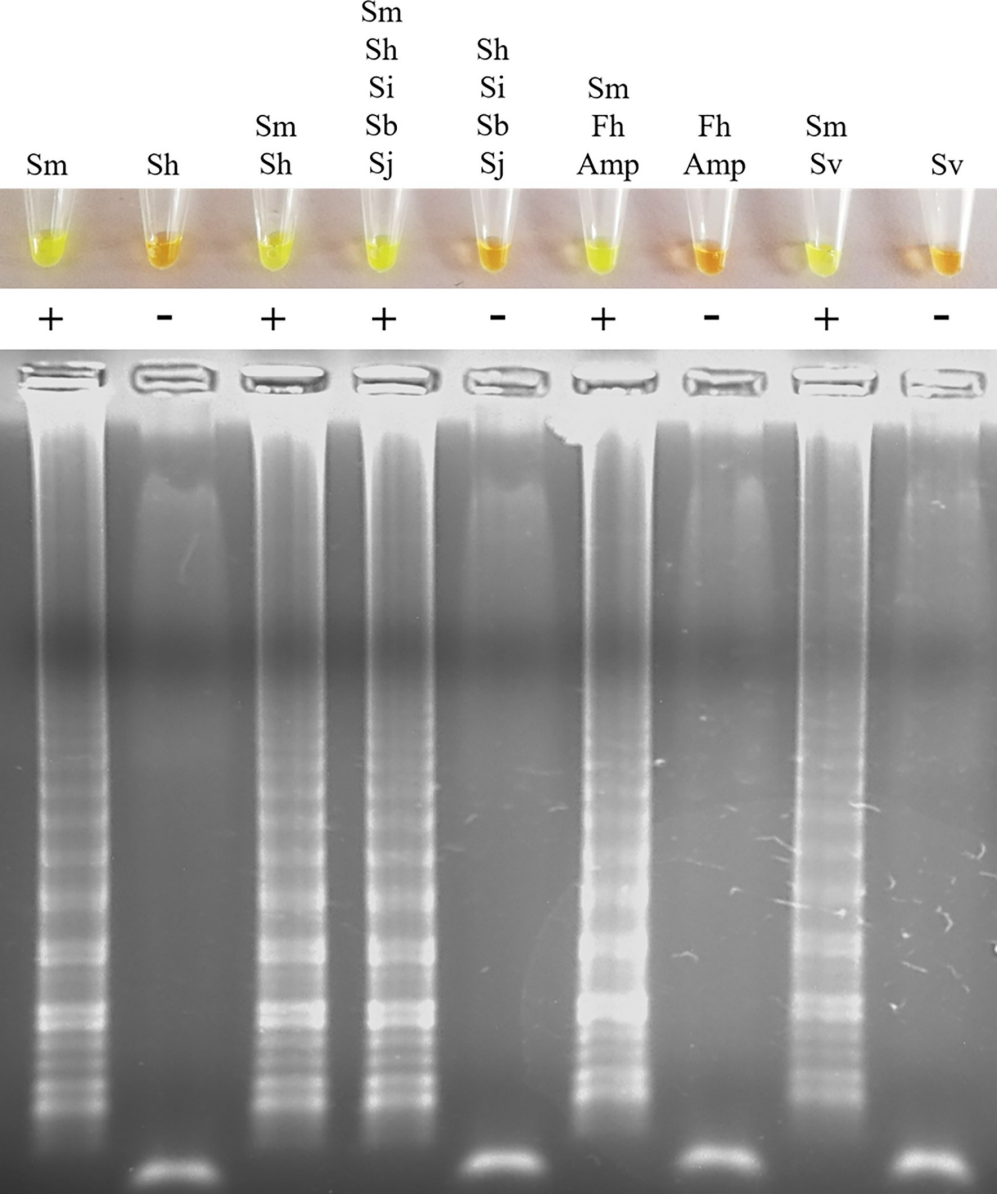
Specificity of the SmMIT-LAMP assay in simulated human urine samples spiked with DNA from several helminths. Figure shows the LAMP results when incubating for 60 min (top, by color change; bottom, by agarose electrophoresis) in urine samples spiked with *S*. *mansoni* DNA alone and in combination with DNA from other helminths. Samples were prepared, each containing 100 μL of urine spiked with 0.5 ng of DNA from each parasite. Sm, *S*. *mansoni*; Sh, *S*. *haematobium;* Si, *S*. *intercalatum;* Sb, *S*. *bovis;* Sj, *S*. *japonicum;* Fh, *Fasciola hepatica;* Amp, *Amphimerus* sp.; Sv, *Strongyloides venezuelensis*.

Fig 2 shows the specificity of the SmMIT-LAMP when incubating for 60 min, which exclusively amplifies DNA from *S*. *mansoni* in simulated situations using urine samples spiked additionally with DNA from several other helminths. Only positive results in urine samples containing DNA from *S*. *mansoni* were obtained. The same results were obtained when incubating for 120 min.

#### Application of SmMIT-LAMP in patient urine samples

**Fig 3:**
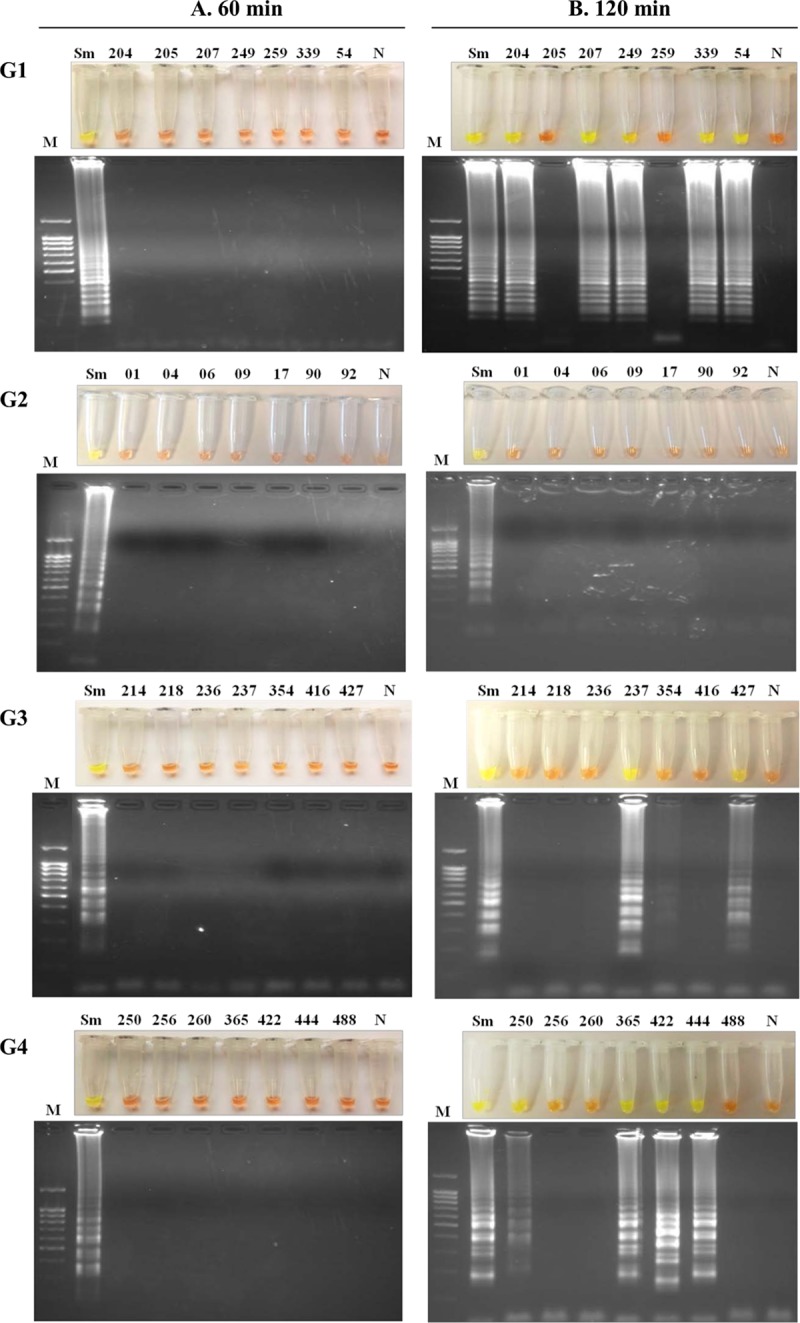
Examination of patient urine samples by SmMIT-LAMP. This figure shows the LAMP results (top, by color change; bottom, by agarose electrophoresis) when analyzing the clinical urine samples by performing the LAMP reaction at 63°C for (A) 60 min or (B) 120 min. G1, G2, G3, G4; groups of urine samples from patients with confirmed infection by *Schistosoma mansoni* (G1), by *S*. *haematobium* (G2), by other nonschistosome parasites (G3) and with eosinophilia, without confirmed diagnosis as well as parasitologically negative (G4). Lanes M, DNA ladder; lanes Sm, positive control (genomic DNA from *S*. *mansoni*; 1 ng); lanes N, negative controls (urine sample from a healthy donor); lanes with numbers, patient urine samples used.

The results of patient urine samples evaluated for *S*. *mansoni* DNA detection using the SmMIT-LAMP assay are shown in Fig 3. When the amplification assays were performed using the standard reaction time of 60 min for SmMIT-LAMP, no positive results were obtained in any group of urine samples (Fig 3A). However, we obtained positive results when using *S*. *mansoni* DNA as a positive control, thus indicating the proper operation of the LAMP assay. As we did not achieve amplification results in clinical samples, we increased the reaction time to 120 min to improve DNA amplification.

In subsequent tests performed for 120 min (Fig 3B), we obtained LAMP-positive results in 5/7 confirmed *S*. *mansoni*-infected urine samples, in 3/7 urine samples with other nonschistosome parasites confirmed parasitologically by microscopy—specifically, three patients infected with *Trichomonas vaginalis* (no. 237), *Enterobius vermicularis* (no. 354) and *Trichuris trichiura* (no. 427)—and in 4/7 urine samples from patients with eosinophilia without a confirmed diagnosis and who were negative for different parasitological tests. No LAMP-positive results in confirmed *S*. *haematobium-*infected urine samples were obtained, thus signifying the specificity of the SmMIT-LAMP. In all assays, the positive *S*. *mansoni* DNA control always amplified, while the negative control (urine sample from a healthy donor) never amplified.

#### Application of SmMIT-LAMP on mouse urine samples

**Fig 4:**
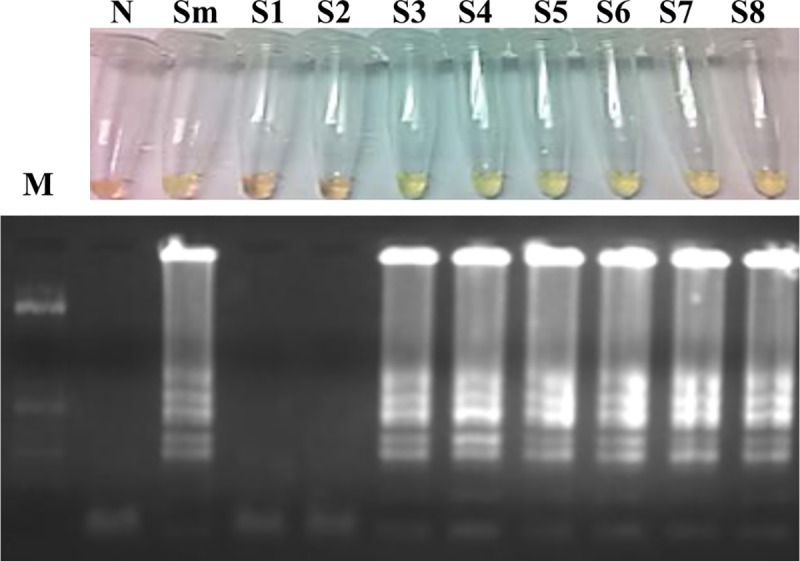
Examination of mouse urine samples by SmMIT-LAMP. Figure shows the 60-min LAMP results (top, by color change; bottom, by agarose electrophoresis) of the analysis of each weekly pool of urine samples obtained from mice infected with 150 cercariae each over a period of 8 weeks. Identical results were obtained for the 120-min LAMP assay. Lane M, DNA ladder; lane N, negative control (urine sample from a noninfected mouse); lane Sm, genomic DNA from *S*. *mansoni* (1 ng); lanes S1-S8, pooled urine samples from individual mice obtained at weeks 1–8 postinfection.

We tested each weekly pool of urine samples obtained from mice infected with *S*. *mansoni* by SmMIT-LAMP (60 min), and positive results were obtained from week 3 p.i. to week 8 p.i. (Fig 4). The positive control was correctly amplified. The pool of urine samples from uninfected mice resulted in a negative LAMP amplification. Identical results were obtained with a reaction time of 120 min.

**Fig 5:**
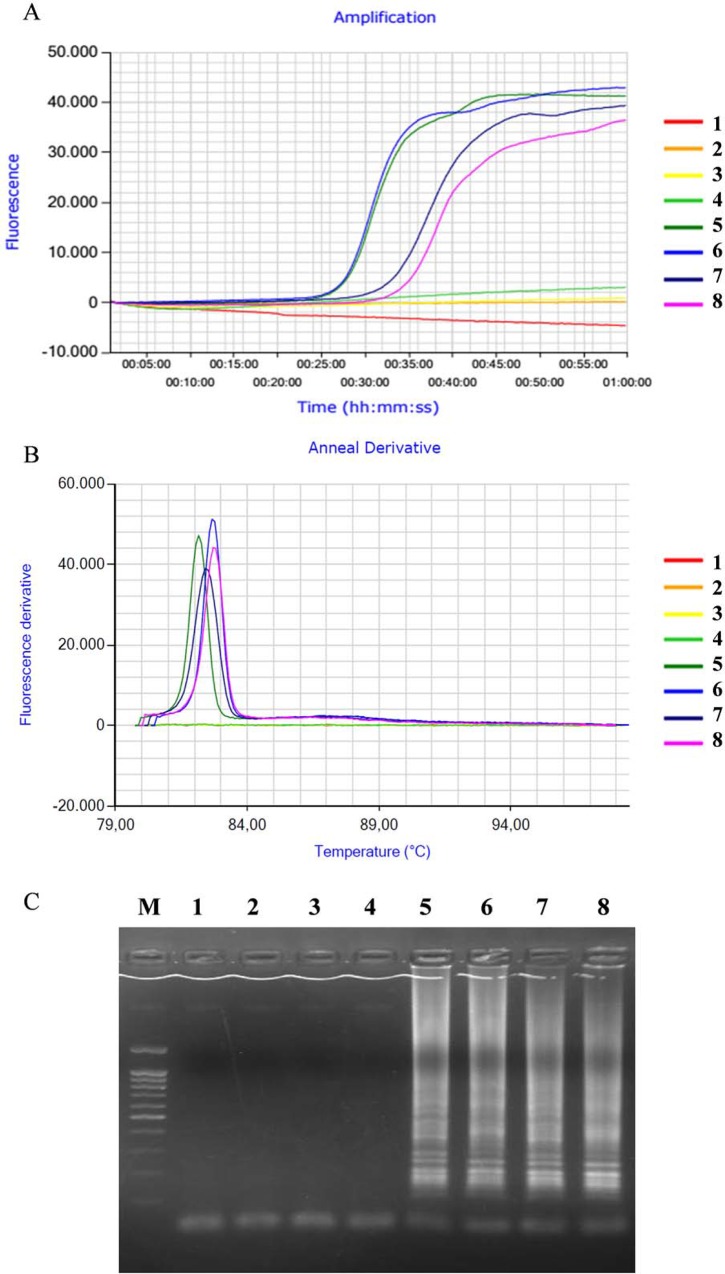
Amplification curves, melt outputs and agarose gel electrophoresis for *S*. *mansoni* in urine samples from experimentally infected mice collected at week 5 p.i. Outputs from the LAMP experimental run showing A) amplification and B) melt outputs using positive and negative samples. C) Agarose gel electrophoresis. Lanes 1 and 2, negative controls (water as template); lanes 3 and 4, negative controls (DNA from uninfected mouse urine as template); lane 5, positive control (*S*. *mansoni* DNA; 1 ng); lanes 6–8, three mouse urine samples. Lane M, DNA ladder.

When the LAMP assay was run in real-time for 60 min at 65°C, DNA extracted from urine collected from three *S*. *mansoni*-infected mice at week 5 p.i. resulted in amplification times of 35:44, 35:29 and 41:14 min, respectively, with melt temperatures ranging between 82.40°C and 82.70°C (Fig 5). Negative controls showed no amplification. Analysis by 1.5% agarose gel electrophoresis confirmed these results.

## Discussion

PCR-based tests in stool specimens have proven more sensitive than microscopy on KK smears for *S*. *mansoni* detection, especially for low worm burdens, such as chronic infection cases [30] and in low transmission areas [31]. Regardless of the test used, the collection of stool samples for diagnostic purposes is usually difficult, and patients perceive that handling stools is dirty and embarrassing [32]. Additionally, the handling, management and storage of large numbers of patient stool samples for diagnosing schistosomiasis can be very laborious in large-scale field trials. In addition, PCR inhibitors are frequently present in stool samples and can lead to false-negative PCR results [33]. Considering the advantages over the stool samples and the proven effectiveness of the PCR-based detection of schistosome-derived DNA in urine, the use of patient urine samples would be an alternative approach for *S*. *mansoni* molecular detection. However, PCR-based technology is not widely used in resource-limited settings because skilled personnel and expensive cyclers are still needed. The inexpensiveness and user-friendliness of the LAMP technique, together with the increasing miniaturization of the isothermal technology as a lab-on-chip, makes it a cost-effective and simple tool that provides an alternative to PCR assays for chip-based point of care (POC) diagnostic applications [34–37]. Even so, at present, the microLAMP systems for POC diagnostics still have limitations and do not completely satisfy World Health Organization (WHO) criteria for equipment- and electricity-free operation [36].

The objective of this work is to determine the feasibility of using urine as a source of DNA in combination with the effectiveness of our LAMP assay for successful *S*. *mansoni* detection in clinical urine samples. First, the sensitivity of the SmMIT-LAMP assay in simulated fresh human urine samples spiked with DNA from *S*. *mansoni* was examined. The detection limits of our LAMP assay were 100 fg/μL when incubating for 60 min and 0.01 fg/μL for 120 min. It has been suggested that a longer incubation time for the LAMP reaction improves the sensitivity, thus avoiding the possibility of obtaining false negatives [38, 39]. Moreover, a reaction time of 120 min has already been successfully used with LAMP for the integrated diagnosis of single or mixed schistosome infection by amplifying *S*. *mansoni* and *S*. *haematobium* DNA from human urine samples [21]. Considering the above, since we first tested the patient urine samples using SmMIT-LAMP for 60 min, and no positive results were obtained, we then increased the incubation time to 120 min and obtained amplification in several urine samples. The DNA of *S*. *mansoni* used as a positive control was always amplified (incubating 60 or 120 min), thus indicating the correct operation of the LAMP assay. Negative controls never amplified, indicating that nonspecific binding due to annealing of primers did not occur. Moreover, we always obtained SmMIT-LAMP-negative results when using incubation times of 60 and 120 min in all parasitologically confirmed *S*. *haematobium*-positive urine samples tested, thus corroborating again that no cross-reaction with that schistosome species occurred [28] and also avoiding potential false-negative results. The possibility of a cross-reaction was completely discarded, as the analysis of urine samples spiked with DNA from other parasites, including several *Schistosoma* species different from *S*. *mansoni*, was negative. Hence, all subsequent SmMIT-LAMP assays to test human clinical samples were performed for 120 min.

The use of frozen storage urine samples collected in a biobank allowed us to select a set of well-defined human samples for the study. We obtained 5/7 (71.4%) SmMIT-LAMP-positive results in those patient urine samples with microscopy-confirmed *S*. *mansoni* infection. However, authentic *S*. *mansoni*-infected samples (nos. 205 and 259) were not amplified. We think that the lack of positive results in these samples cannot be due to the lack of a proper operation of the SmMIT-LAMP, given the high sensitivity shown by the method, which worked properly with the other five microscopy-confirmed *S*. *mansoni* samples. It is possible that several other factors, such as bacterial and/or fungal contamination during the storage of urine samples, long-term frozen storage conditions without a preserving solution or repetitive freeze-thaw cycles for different trials, could be the reason for the lack of *S*. *mansoni* DNA detection. In a previous work, we described similar complications in obtaining positive results in *S*. *mansoni* DNA detection when 18 patient urine samples infected with the parasite were tested by PCR [40]. However, it must be considered that we have now achieved a greater number of positive results by SmMIT-LAMP than using PCR for *S*. *mansoni* DNA detection in urine samples. This could be due to the increased sensitivity and the greater robustness of LAMP over PCR-based methods for diagnostic applications [41, 42]. Nevertheless, because the patients themselves often collect their urine samples outside of a medical setting, molecular analysis can be very susceptible to preanalytical contamination issues. In this sense, it would be interesting to identify reagents that stabilize cell-free DNA in urine specimens, allowing convenient sample collection, transport and storage for further molecular analysis.

Regarding the SmMIT-LAMP-positive results obtained from patient urine samples with parasitologically confirmed infection with nonschistosome parasites, i.e., *Trichomonas vaginalis* (no. 237), *Enterobius vermicularis* (no. 354) and *Trichuris trichiura* (no. 427), it is not uncommon in endemic areas to find patients coinfected with schistosomes and other organisms, such as bacteria, protozoa and helminths [43]. We think that these results could be authentic microscopically undetected *S*. *mansoni* infections and not likely due to cross-reactions, as we obtained a SmMIT-LAMP-negative result in another sample (no. 416) with a microscopy-confirmed infection with *T*. *trichiura*, among other parasites.

We also obtained SmMIT-LAMP-positive results in several patient urine samples with eosinophilia but without a confirmed diagnosis and who were negative for any parasite in microbiological tests. Eosinophilia is usually used as a biomarker for helminthic infections, including schistosomiasis [44], but it is not predictive of *Schistosoma* species infection and can lead to inconsistent results [45]. The SmMIT-LAMP-positive results obtained could be authentic *S*. *mansoni* infections that were microscopically undetected because of the classically low sensitivity of the parasitological diagnosis (especially in low-intensity infections) or affected by day-to-day variability in egg excretion [46]. Another explanation could be a detection of a prepatent schistosome infection.

The viability of the SmMIT-LAMP assay for detection of *S*. *mansoni*-derived DNA in urine samples was also tested in a mouse model of schistosomiasis. We obtained positive results in pooled samples collected from week 3 to week 8 p.i., suggesting that an early diagnosis of active *S*. *mansoni* infection is feasible using urine samples and a LAMP assay, as previously reported using mouse feces [28]. Since the mouse urine studies were on pooled samples, there was no way to determine a potential false-negative rate for these samples. Consequently, we tested fresh urine samples collected *ad hoc* at week 5 p.i. from three experimentally *S*. *mansoni*-infected mice by a real-time SmMIT-LAMP, and we individually verified that amplifications began after 35 min of incubation at 65°C. These positive results in fresh mouse urine samples could explain the failure in amplification in stored frozen microscopy-confirmed *S*. *mansoni* clinical urine samples, possibly due to contamination prior to storage or DNA damage hindering proper amplification, as previously suggested. Therefore, a longer incubation time for a conventional SmMIT-LAMP reaction allowed us to improve the efficiency of the amplification, thus avoiding false negatives in parasitological *S*. *mansoni*-confirmed infections and in other clinical urine samples analyzed. Thus, the successful detection of *S*. *mansoni* DNA in long-term frozen stored and likely partially degraded urine samples analyzed makes our SmMIT-LAMP assay a potentially robust tool for molecular diagnosis in low-complexity laboratories, mainly in endemic areas of schistosomiasis. However, further studies are needed using inexpensive, easy and good-quality DNA from urine samples to reduce the costs of the analysis and to reduce the amplification time required, especially if unpreserved urine samples are used. Additionally, it would be ideal to increase the number of urine samples to examine how reproducible the technique is when testing in field settings.

The preliminary results presented here suggest that our SmMIT-LAMP assay could be a promising molecular tool applicable to patient urine samples to diagnose intestinal schistosomiasis caused by *S*. *mansoni*.

## Supporting information

S1 Flow chart (PDF)
