# Supplementary figures and images for: Detection of Schistosoma mansoni-derived DNA in human urine samples by loop-mediated isothermal amplification (LAMP)
Source: PLoS One. 2019 Mar 26;14(3):e0214125. doi: 10.1371/journal.pone.0214125 (PMC6435178; doi:10.1371/journal.pone.0214125)

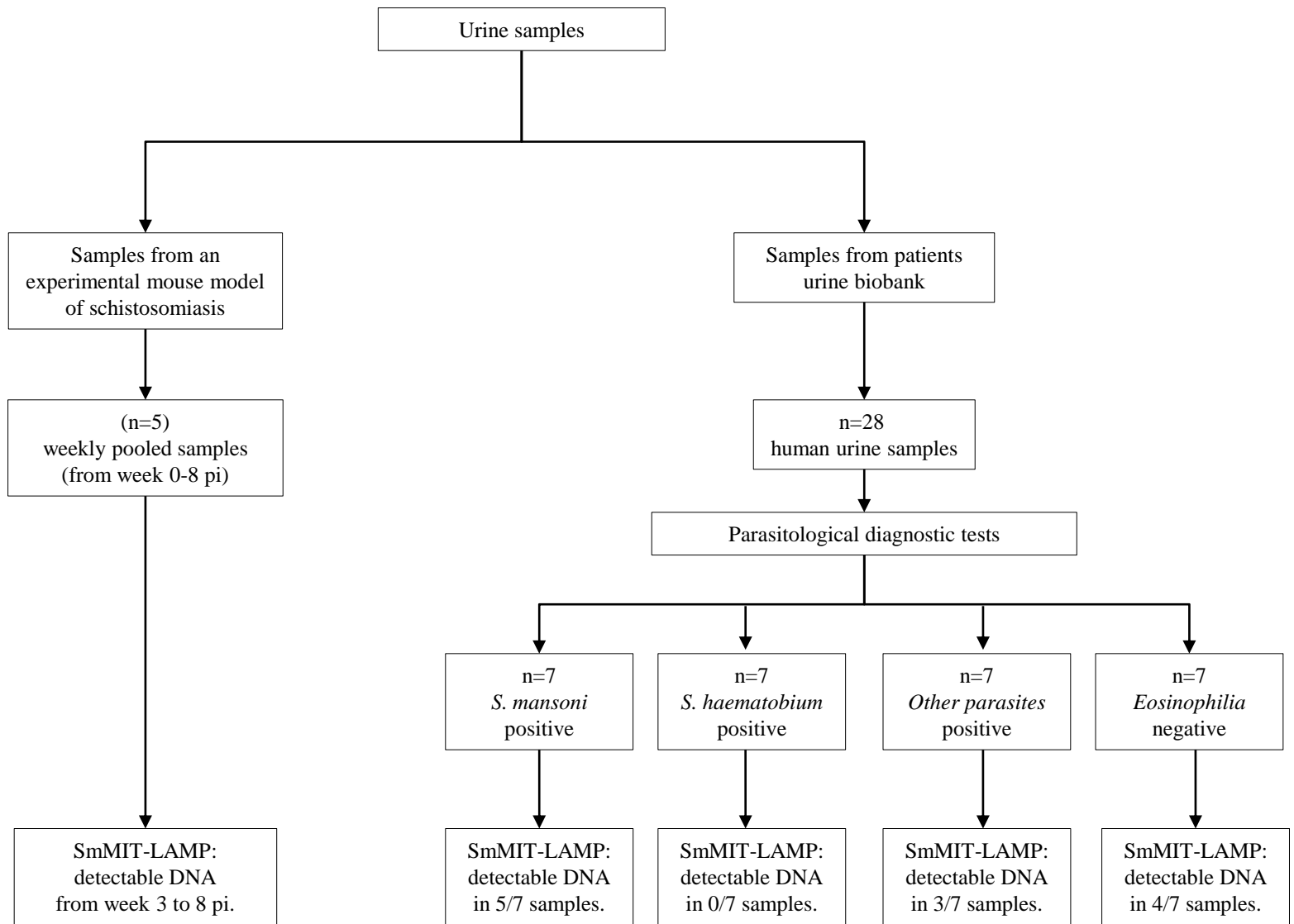

Supplement: S1 Flow chart — (PDF) [file pone.0214125.s001.pdf]
